# Supplementary material for: Improving neglected tropical disease services and integration into primary healthcare in Southern Nations, Nationalities and People’s Region, Ethiopia: Results from a mixed methods evaluation of feasibility, acceptability and cost effectiveness
Source: PLoS Negl Trop Dis. 2025 Feb 20;19(2):e0011718. doi: 10.1371/journal.pntd.0011718 (PMC11870372; doi:10.1371/journal.pntd.0011718)
Supplement: S1 Table — (DOCX) [file pntd.0011718.s001.docx]

Table 1. Characteristics of patients enrolled in the baseline and endline assessment.

| Variable |  | Baseline | Endline |
| --- | --- | --- | --- |
| *Health facility type* | Hospital | 10 (23%) | 45 (34.9%) |
|  | Health centre | 21 (49%) | 58 (45%) |
|  | Health post | 12 (28%) | 26 (20.2%) |
|  | | | |
| *Sex* | Female | 23 (53.5%) | 74 (57.4%) |
|  | Male | 20 (46.5%) | 55 (42%) |
|  | | | |
| *Age category* | <5 | 4 (9.3%) | 18 (14%) |
|  | 5-15 | 2 (4.7%) | 11 (8.5%) |
|  | 15+ | 37 (86%) | 100 (77.5%) |
| Total |  | 43 | 129 |
